# Combination of *Clostridium butyricum* and Corn Bran Optimized Intestinal Microbial Fermentation Using a Weaned Pig Model

**DOI:** 10.3389/fmicb.2018.03091

**Published:** 2018-12-18

**Authors:** Jie Zhang, Jian Sun, Xiyue Chen, Cunxi Nie, Jinbiao Zhao, Wenyi Guan, Lihui Lei, Ting He, Yiqiang Chen, Lee J. Johnston, Jinshan Zhao, Xi Ma

**Affiliations:** ^1^State Key Laboratory of Animal Nutrition, College of Animal Science and Technology, China Agricultural University, Beijing, China; ^2^Department of Animal Husbandry and Veterinary, Beijing Vocational College of Agriculture, Beijing, China; ^3^College of Animal Science and Technology, Shihezi University, Xinjiang, China; ^4^West Central Research and Outreach Center, University of Minnesota, Morris, MN, United States; ^5^College of Animal Science and Technology, Qingdao Agricultural University, Shandong, China; ^6^Department of Internal Medicine, University of Texas Southwestern Medical Center, Dallas, TX, United States; ^7^Department of Biochemistry, University of Texas Southwestern Medical Center, Dallas, TX, United States

**Keywords:** synbiotics, *Clostridium butyricum*, corn bran, intestinal bacteria, short chain fatty acids, weaned pig model

## Abstract

Experimental manipulation of the intestinal microbiota influences health of the host and is a common application for synbiotics. Here *Clostridium butyricum* (*C. butyricum*, *C.B*) combined with corn bran (*C.B* + Bran) was taken as the synbiotics application in a waned pig model to investigate its regulation of intestinal health over 28 days postweaning. Growth performance, fecal short chain fatty acids (SCFAs) and bacterial community were evaluated at day 14 and day 28 of the trial. Although the *C.B* + Bran treatment has no significant effects on growth performance (*P* > 0.05), it optimized the composition of intestinal bacteria, mainly represented by increased acetate-producing bacteria and decreased pathogens. Microbial fermentation in the intestine showed a shift from low acetate and isovalerate production on day 14 to enhanced acetate production on day 28 in the *C.B* + Bran treatment. Thus, *C.B* and corn bran promoted intestinal microbial fermentation and optimized the microbial community for pigs at an early age. These findings provide perspectives on the advantages of synbiotics as a new approach for effective utilization of corn barn.

## Introduction

Great attention has been paid to the important influences of dynamic microbial communities on human health. Several animal studies have proven that experimental manipulations of the intestinal microbiota can modify many aspects of the host’s health. Commonly, probiotics have been applied to manipulate the intestinal microbiota. *Clostridium butyricum* (*C. butyricum, C.B*) is an anaerobic, gram-positive bacillus found in the intestine of healthy animals and is commonly considered as a kind of probiotics. *C.B* plays an important role in optimizing the intestinal microbial community, especially in the colon, and maintains the harmonious intestinal microecology by inhibiting proliferation of harmful bacteria (Howarth and Wang, 2013). *C.B* prefers dietary fiber, which is not digested directly by enzymes of monogastric animals, as its fermentable substrate (Sawicki et al., 2017). Orally administered *C.B* spores germinate and grow in intestinal tracts and produce a mass of short chain fatty acids (SCFAs) including acetate, propionate and butyrate (Sun et al., 2016; Jia et al., 2017) by fermenting non-digestible polysaccharides (Chen et al., 2013). SCFAs are major anions in the colon that are absorbed rapidly and stimulate absorption of water and sodium. SCFAs can be oxidized to serve as fuels for colonic cells. Among them, butyrate is the main end products of *C.B* (Cassir et al., 2016) and the major nutrient for energy metabolism (Jackie et al., 2013; van der Beek et al., 2017) of colonic epithelial cells (Ma et al., 2012; Gonçalves and Martel, 2013; Simeoli et al., 2017). SCFAs can also decrease colonic pH, stimulate intestinal peristalsis, improve the intestinal micro-environment and regulate the micro-ecological balance of the colon (Ma et al., 2018). In addition, SCFAs have an important role to play in proliferation and differentiation of colonocytes and regulation of gene expression in colonic epithelial cells (Nicholson et al., 2012). Theoretically, diets containing *C.B* could beneficially impact growth performance, SCFAs formation, and stability of microbial community in the gut of weaned pigs.

The combination of probiotics and fibrous prebiotics is called synbiotics. Dietary fiber includes a soluble part (SDF) and an insoluble part (IDF), and the later mainly consists of cellulose, hemicellulose, and lignin (Brownlee, 2011). In the past, weaned animals were considered unable to ferment carbohydrates. Recent research results suggest a proper addition of dietary fiber can enhance intestinal health, modulate the microbial community and support innate immunity of intestinal mucosa in weanling piglets (Han et al., 2017). Previous studies have proven that dietary fiber exerts its function by forming SCFAs from fermentation of saccharolytic microbiota, especially cellulose-degrading ones (Chen T. et al., 2017). An addition of dietary fiber can serves as the fermentation substrate of hindgut microorganisms and improves intestinal health by modulating gut microbial composition and function (Jeffery and O’Toole, 2013; Desai et al., 2016; Martens, 2016; Brahma et al., 2017). The intestinal microbiota contains highly diverse communities and has multiple roles in metabolism and health of the host (Chen et al., 2015; Wang et al., 2016).

As the main by-product of corn processing, corn bran is used widely as an ingredient for animal feed. Corn barn has the highest content of dietary fiber among all cereal brans (Liu et al., 2017). However, the high content of plant polysaccharides in corn bran limits its nutritive value for pigs. Several processing technologies such as solid-state fermentation, have been applied to corn barn as an effort to improve nutritive value (Liu et al., 2017). Use of saccharolytic bacteria might be another approach to enhance the nutritive value of corn barn. The combination of *C.B* and corn bran might be used as an effective synbiotics. Synbiotics are a mixture of probiotics and prebiotics that can exert the biogenic activity of probiotics, but also selectively increase the number of bacteria, making the probiotics more effective and lasting (Duncan and Flint, 2013). Thus, this experiment was conducted to compare the influences of synbiotics with *C.B* and corn bran or a single addition of *C.B* on intestinal health using a weaned piglet model. A long-term objective of this research is to evaluate the utility of synbiotics in improving the nutritional value of low quality, fibrous feed stuff.

## Materials and Methods

### Ethics Approval and Consent to Participate

All procedures of this experiment were approved by the animal protection and utilization organization committee of China Agricultural University (CAU20171015-3).

### Pigs, Diets, and Experimental Protocol

Newly weaned pigs (*n* = 48; Landrace × Large White) were picked from 24 litter piglets at 28 day age. Pigs (8.09 ± 0.25 kg) were allotted randomly to a basal diet with 1% *C.B* or the basal diet with 1% *C.B* and 5% corn bran (*C.B* + Bran). One pen as a replicate, four replicates per treatment and six pigs per replicate. The standard corn-soybean basal diet was formulated based on the standard ileal digestible amino acids to satisfy 11–20 kg pigs’ requirement (NRC, 2012. See Table 1).

**Table 1:** Ingredient composition and nutrient content of experimental diets (%, Dry matter basis)^1^.

Items	*C.B*	*C.B* + Bran
**Ingredients**		
Corn	55.02	50.12
Soybean meal	15.70	15.30
EFFSB	5.00	5.00
Corn bran	–	5.00
*C. butyricum*	1.00	1.00
Soybean protein concentrate	4.00	4.00
Fish meal	4.00	4.00
Whey powder	8.00	8.00
Sucrose	3.00	3.00
Zinc oxide	0.28	0.28
Soya-bean oil	1.30	1.50
Calcium hydro phosphate	1.20	1.20
Limestone	0.50	0.50
Salt	0.30	0.30
L-Lys HCl	0.30	0.35
Met	0.20	0.25
Thr	0.15	0.18
Trp	0.10	0.10
Val	0.20	0.25
Chromic oxide	0.25	0.25
Premix^2^	0.50	0.50
Total	100.00	100.00
**Nutrient concentration**		
DE, MJ⋅kg^-1^	14.50	14.50
CP	18.50	18.50
NDF	11.75	13.25
ADF	4.25	4.80
Ca	0.80	0.80
P	0.60	0.60
SID Lys	1.30	1.30
SID Met + Cys	0.80	0.80
SID Thr	0.90	0.90
SID Trp	0.30	0.30


*^1^ADF, acid detergent fiber; C.B, basal diet + C. butyricum; C.B + Bran, basal diet + C. butyricum + corn bran; CON, basal diet; CP, crude protein; DE, digestible energy; EFFSB, extruded full fat soybeans; NDF, neutral detergent fiber; SID, standard ileal digestibility; Lys, lysine; Met, methionine; Thr, threonine; Trp, tryptophan; Val, valine; Ca, calcium; P, phosphorus. ^2^Supplied per kilogram of complete diet: vitamin A, 12,000 IU; vitamin D_3_, 2,500 IU; vitamin E, 30 IU; vitamin K_3_, 3 mg; vitamin B_12_, 0.012 mg; riboflavin (vitamin B_2_), 4 mg; niacin (vitamin B_3_), 40 mg; pantothenic-acid (vitamin B_5_), 15 mg; choline chloride, 400 mg; folacin, 0.7 mg; thiamine (vitamin B_1_), 1.5 mg; (vitamin B_6_), 3 mg; biotin, 0.1 mg; Zn, 100 mg as ZnO; Mn, 40 mg; Fe, 90 mg; Cu, 200 mg; I, 0.35 mg; Se, 0.3 mg.*

The *C.B* supplement (China Microorganism Preservation Center, Strain No. 1.336) was included at 1% and consisted of 1 × 10^8^ CFU/g in spore state.

Each animal was weighed on days 14 and 28 of the trial and feed intake was recorded weekly for every pen. ADFI, ADG, and F/G were calculated. Fresh fecal samples from 8 pigs per treatment were collected and immediately frozen in liquid nitrogen on day 14 and day 28. Fecal samples were stored at -80°C for bacterial DNA and bacterial metabolite analysis.

### Extraction of Fecal DNA

E.Z.N.A Stool DNA Kit (Omega Bio-Tek Inc., United States) was used following the manufacturer’s protocols to detect total bacterial DNA in fecal samples. A nanodrop 2000 spectrophotometer (Thermo Fisher Scientific, United States) was used for DNA micro-quantification and 1% agarose gel electrophoresis was used for detection of DNA size fragments. Finally, quantified DNA was kept at -20°C for DNA sequencing analysis.

### Polymerase Chain Reaction (PCR) Amplification

Amplification of V3–V4 regions of the bacterial 16S rRNA gene was accomplished via TransStart Fastpfu^®^ DNA Polymerase (Takara, Japan) and a PCR procedure. The upstream primer was 5′-barcode-ACTCCTACGGGAGGCAGCA-3′ and the downstream primer was 5′GGACTACHVGGGTWTCTAAT-3′. The reaction system of PCR (20 μL) include 5 × FastPfu buffer, 4 μL; 2.5 mM dNTPs, 2 μL; each primer (5 μM), both 1.6 μL; FastPfu polymerase, 0.4 μL and template DNA, 10 ng. The PCR procedure included 95°C denaturation for 3 min; then 26 cycles with 95°C for 30 s, 55°C for 30 s, and 72°C for 45 s and finally 72°C for 10 min.

### Illumina MiSeq Sequencing

After purification with the AxyPrep DNA Purification kit (Axygen Biosciences, United States), PCR products were detected by Agarose gel (2%) electrophoresis and were quantified using PicoGreen dsDNA Quantitation Reagent (Invitrogen, United States) on QuantiFluor-ST Fluorometer (Promega, United States). After that, collected amplicons for paired-end sequencing (2 × 300 bp) according to standard protocols. This process was completed on the Illumina MiSeq platform (Allwegene, China). The raw data in this manuscript have been uploaded to the NCBI SRA Database under an accession no. SRP159591.

### Bioinformatics Analysis of Sequencing Data

For raw fastq files analysis, the first step was to demultiplex and quality-filter data via QIIME (version 1.17). basic principles used in this process were: (i) Sequencing reads were trimmed at the sites with an average quality score <20 over a 50 bp sliding window and deleted trimmed reads less than 50 bp; (ii) The reads that contained mismatching barcode were deleted; and (iii) Removing the paired reads with less than 10 bp overlapping.

**FIGURE 1:**
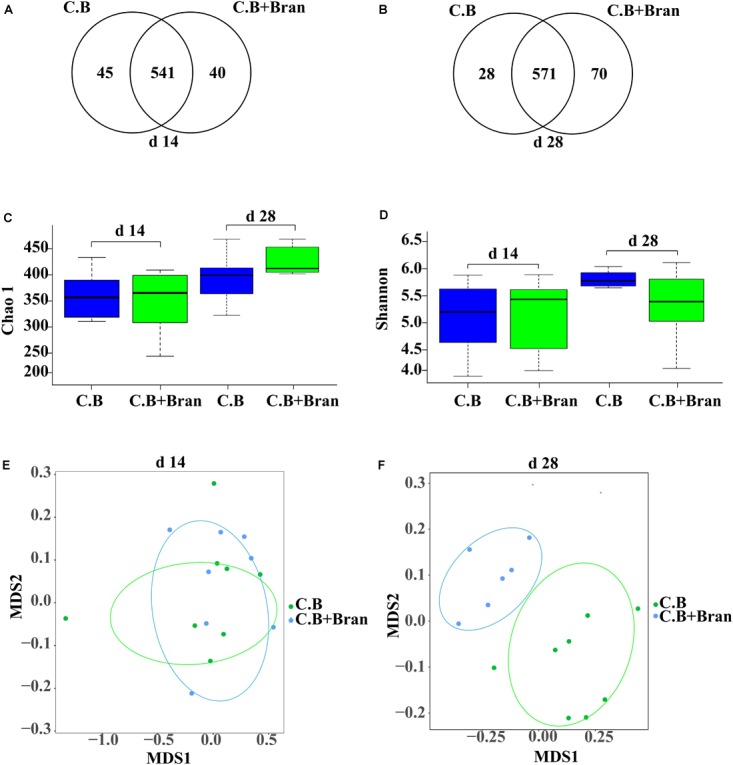
Richness, diversity, and similarity of bacterial communities between different dietary treatments in weaned pigs. Venn diagram of the OTUs in basal diet with *C. butyricum* (*C.B*) group and basal diet with the combination of *C. butyricum* and corn bran (*C.B* + Bran) group at the 14th day **(A)** and 28th day **(B)** after weaning. Bacterial richness was estimated by the Chao1 value **(C)**. Bacterial diversity was estimated by Shannon index **(D)**. The diff-NMDS plot comparative analysis of sample in bacterial community between two groups were showed on day 14 **(E)** and 28 **(F)** after weaning.

**FIGURE 2:**
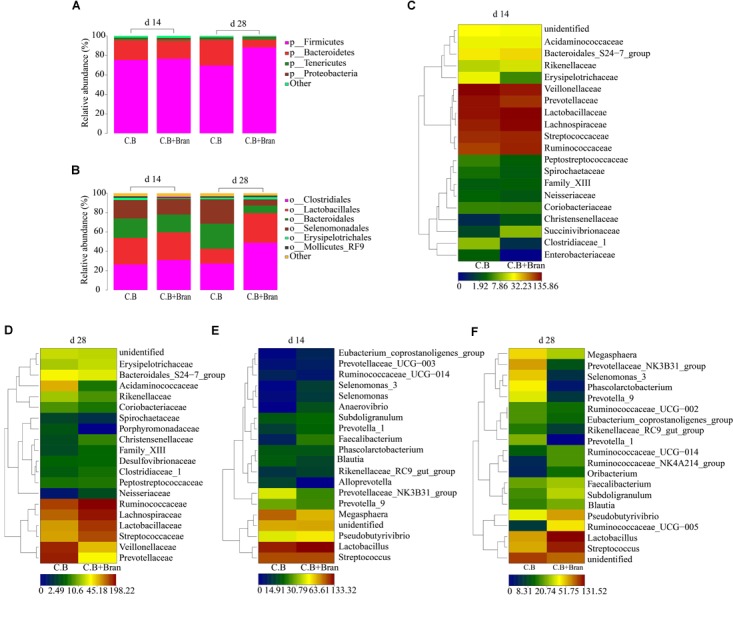
Community structures of fecal bacteria between different dietary treatments in weaned pigs. Bacterial community structure in basal diet with *C. butyricum* group (*C.B*) and basal diet with the combination of *C. butyricum* and corn group (*C.B* + Bran) were described at the phylum **(A)**, order **(B)**, family **(C,D)**, and genus **(E,F)**.

**FIGURE 3:**
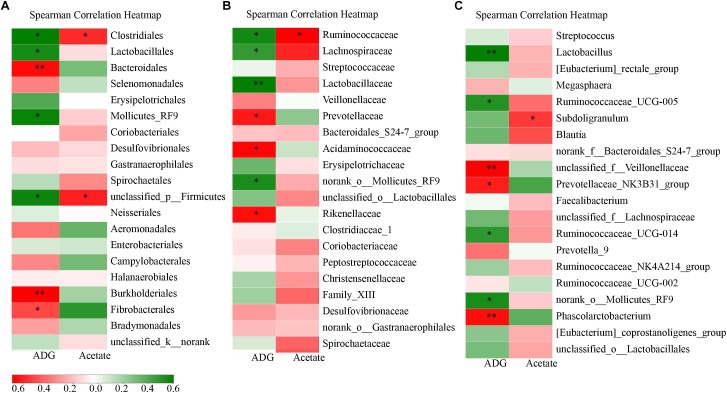
Correlation analysis between the varied ADG and fecal acetate and corresponding intestinal flora at different levels on d 28. **(A)** is the order level. **(B)** is the family level. **(C)** is the genus level. Star in red color bar means that there is a significant positive correlation. Star in green bar means that there is a significant negative correlation.

UPARSE (version 7.1^1^) was used to gather OTUs with a 97% similarity. UCHIME was used to identify and delete chimeric sequences. RDP Classifier^2^ based on Silva (SSU115) 16S rRNA database was used to complete the taxonomic analysis for each 16S rRNA gene sequence with a confidence threshold of 70%. Venn diagrams software of R tools generated Venn figures (Figures 1A,B), which represented visually of the similarity and overlap of the OTU samples. The alpha diversity indexes, including Chao index and Shannon index, were all calculated using qiime software (version v.1.8^3^) of Mothur v.1.21.1 and produced Figures 1C,D. Vegan and ggplot2 package of R tools conducted the Non-metric multidimensional scaling (NMDS) analysis and produce Figures 1E,F. Based on the results of taxonomic analysis, using R tool to produce the diagram of species composition in different samples (Figures 2A,B). To clustering data for abundance similarity between species or samples, using vegdist and hclust of vegan package of R tools to do distance calculation and clustering analysis, which distance algorithm did by Bray-Curtis and clustering method did by complete-linkage. Diagram of results shown as Figures 2C–F, 3.

### Detection of SCFAs

About 0.5 g feces were put into a 10 mL polypropylene tube and diluted with 8 mL deionized water. Tubes containing samples rested in an ultrasonic bath for 30 min, and were centrifuged at 8,000 rpm for 10 min. The supernatant was drained into an empty tube and diluted 50 times and then filtered with a 0.22 μm filter. High performance ion chromatography of ICS-3000 (Dionex, United States) was used to analyze the components of 25 μL of extracted sample solution. Separate organic acids used AS11 analytical column (250 mm × 4 mm); separate the other gradient conditions used an AG11 guard column. Varying concentrations of potassium hydroxide was used for gradient contrast. Those concentrations were: 0.8–1.5 mM for 0–5 min; 1.5–2.5 mM for 5–10 min; 2.5 mM for 10–15 min. The flow rate is 1.0 mL/min.

### Statistical Analysis

The data analysis and graphic analysis of growth performance and organic acid data were performed by unpaired *t*-test of SPSS 19.0 and GraphPad prism 6.0. Results are shown as means ± SEMs. *P*-value <0.05 was considered a significant difference.

## Results

### Effect of *C.B* and Corn Bran on Growth Performance

From the day 0 to 14 and day 14 to 28, growth performance indicated by average daily feed intake (ADFI), average daily gain (ADG) and the ratio of ADFI to ADG (F/G) between two treatments showed no significant difference (Table 2).

**Table 2:** Effect of dietary *C.B* and *C.B*+ corn bran inclusion on weaned pigs growth performance^1^.

Items	Day 0–14			Day 14–28		
	*C.B*	*C.B* + Bran	*P*-value	*C.B*	*C.B* + Bran	*P*-value
ADFI, g	464.5 ± 42.2	462.7 ± 40.1	>0.05	678.7 ± 34.9	641.4 ± 31.0	>0.05
ADG, g	292.1 ± 45.2	304.8 ± 27.4	>0.05	435.7 ± 18.5	415.5 ± 3.1	>0.05
F/G	1.65 ± 0.14	1.53 ± 0.08	>0.05	1.57 ± 0.13	1.54 ± 0.07	>0.05


*^1^Values are means ± SEMs, n = 24/treatment. ADFI, average daily feed intake; ADG, average daily gain; C.B, basal diet + C. butyricum; C.B + Bran, basal diet + C. butyricum+ corn bran; F/G, the ratio of ADFI to ADG.*

### Effects of *C.B* and Corn Bran on Intestinal Bacterial Richness, Diversity, and Similarity

To understand changes in intestinal bacteria, we performed 16S rRNA gene sequencing of fecal samples on day 14 and 28 after weaning. After quality control, size filtering, and chimera removal, 449,014 and 463,345 clean reads were obtained from feces collected on day 14 and day 28, respectively. The total operational taxonomic units (OTU) numbers were classified at 97% similarity, with 626 OTUs and 669 OTUs detected in fecal samples on days 14 and 28, respectively. Fecal bacterial communities of the two groups shared about 86.42% on day 14 and 85.35% on day 28 (Figures 1A,B). Interestingly, the number of unique OTUs in the *C.B* + Bran treatment was well above that in *C.B* group on day 28.

The Chao1 index and Shannon index were detected to study the effect of *C.B* and corn bran inclusion on bacterial abundance and diversity. Between *C.B* and *C.B* + Bran groups, no significant differences were observed on both day 14 and 28 (Figures 1C,D). The β diversity of OTU community comparisons done by hierarchical clustering showed no differences between the two groups on day 14 (Figure 1E). But on day 28, intestinal microbiota of two treatments were clustered separately (Figure 1F), indicating a significant effect of corn bran in the later period of the experiment.

### Effects of *C.B* and Corn Bran on Community Structure of Fecal Bacteria

The most prevalent phyla were Firmicutes and Bacteroidetes in the present fecal samples, accounting for more than 95% of the total microbiota (Figure 2A). On day 14 after weaning, no significant differences were found in the dominant phyla among the two treatments. On day 28, the proportion of Firmicutes dramatically increased from 69.67% in the *C.B* group to 88.14% in the *C.B* + Bran group, while the proportion of Bacteroidetes sharply decreased from 25.72 to 7.83%.

At the order level, Firmicutes were mainly composed of Clostridiales, Lactobacillales, and Selenomonadales, while Bacteroidales was the dominant order of Bacteroidetes (Figure 2B). Erysipelotrichales of Firmicutes decreased significantly in the *C.B* + Bran group on day 14. On day 28, Clostridiales and Lactobacillales increased dramatically from 28.37 to 48.91% and 14.94 to 30.46%, respectively in the *C.B* + Bran group. However, Selenomonadales of Firmicutes dropped its proportion significantly from 24.53% in the *C.B* group to 6.49% in the *C.B* + Bran group on day 28. Bacteroidales as the predominant order of Bacteroidetes were markedly lower in the *C.B*+ Bran group on day 28.

At the family level, the only change on day 14 occurred in the proportion of Erysipelotrichaceae that declined from 2.27% in the *C.B* group to 0.61% in the *C.B* + Bran group (*P* < 0.05) (Figure 2C). On day 28, changes between the two groups were multiple and various (Figure 2D). In the order of Clostridiales, Ruminococcaceae and Lachnospiraceae increased by 9% (*P* < 0.05), while Veillonellaceae and Acidaminococcaceae decreased from 18.18 and 6.35% to 5.86 and 0.64%, respectively (*P* < 0.05) in the *C.B* + Bran group compared with the *C.B* group. Additionally, Prevotellaceae showed a similar significant decrease with its order Bacteroidales.

Genera in fecal samples on day 14 displayed slight changes with increased *Lactobacillus* and decreased *Megasphaera* in the *C.B* + Bran group without difference (Figure 2E). On day 28, there was no significant difference in the dominant genera including *Lactobacillus* and *Streptococcus*. *Prevotellaceae_NK3B31_group*, *Prevotella_9* and *Prevotella_1* of Prevotellaceae, as well as *Ruminococcaceae_UCG_005* of Ruminococcaceae changed resembled to their change in family level (*P* < 0.05) (Figure 2F).

### Effects of *C.B* and Corn Bran on Concentration of Fecal SCFAs

To evaluate the effect of combining *C.B* with corn bran on intestinal fermentation, the concentration of fecal SCFAs, including acetate, propionate, butyrate, isobutyrate, and isovalerate were measured (Table 3). On day 14, concentration of acetate and isovalerate were lower (*P* < 0.05) in the *C.B* + Bran group than the *C.B* group. On day 28, the concentration of acetate increased with the combined addition of *C.B* and corn bran compared with the single addition of *C.B* (*P* < 0.05).

**Table 3:** Effect of dietary *C.B* and *C.B* + corn bran inclusion on concentration of fecal SCFAs (mg/g feces)^1^.

Items	Day 14			Day 28		
	*C.B*	*C.B* + Bran	*P*-value	*C.B*	*C.B* + Bran	*P*-value
Acetate	3.11 ± 0.21^a^	2.69 ± 0.23^b^	<0.05	2.91 ± 0.14^b^	3.46 ± 0.15^a^	<0.05
Propionate	2.02 ± 0.14	1.57 ± 0.17	>0.05	2.10 ± 0.16	2.17 ± 0.13	>0.05
Butyrate	0.97 ± 0.28	0.81 ± 0.18	>0.05	1.14 ± 0.11	1.26 ± 0.10	>0.05
Isovalerate	0.15 ± 0.03^a^	0.07 ± 0.05^b^	<0.05	0.16 ± 0.02	0.17 ± 0.03	>0.05
Total acid	6.93 ± 0.54	5.69 ± 0.78	>0.05	6.73 ± 0.48	7.76 ± 0.46	>0.05


*^1^Values are means ± SEMs, n = 8/treatment. Different superscripts in same row mean a significant difference, P < 0.05. SCFAs, short chain fatty acids; C.B, basal diet + C. butyricum; C.B + Bran, basal diet + C. butyricum + corn bran. ^a,b^ Different superscript within a row means significantly different (P < 0.05).*

### Correlation Analysis Between the Varied Index (Growth Performance and Fecal SCFAs) and Corresponding Intestinal Flora

To further discover whether the effects of *C.B* with corn bran on the intestinal microbiota were associated with the fluctuating growth performance and fecal SCFAs, the correlation analysis between the differentially abundant intestinal bacteria at the order, family and genus level and ADG and acetate on day 28 was completed. The community abundance of the orders Clostridiales, Lactobacillales and Bacteroidales were correlated negatively with ADG on day 28 (Figure 3). Down to the family and genus level, Ruminococcaceae with its genus (*Ruminococcaceae_UCG-005*, *Ruminococcaceae_UCG-014*) and Lachnospiraceae in the order Clostridiales as well as *Lactobacillus* of Lactobacillaceae in the order Lactobacillales were correlated negatively with ADG on day 28. However, Bacteroidales including *Prevotellaceae_NK3B31_group* and *Phascolarctobacterium*, as well as Burkholderiales and Fibrobacterales were correlated positively with ADG on day 28. For the increased fecal acetate, the genus *Subdoligranulum* of Ruminococcaceae in Clostridiales was correlated positively with it on day 28.

## Discussion

Previous researches on the effects of corn bran on body health varied according to many factors with some of them indicating that fiber-rich diets would enhance growth performance (Gerritsen et al., 2012) while others showed reduced or unchanged digestibility of nutrients and energy (Jaworski et al., 2017; Morowitz et al., 2017). Dietary factors including source, solubility, processing and dose (Williams et al., 2017) can affect intestinal fermentation. Considering the low utilization of corn bran, it is necessary to link corn barn with new treatments such as combining with probiotics that will improve nutritional value. Thus, this manuscript aimed at investigating the effect of adding *C.B* and corn bran for intestinal health via using a weaned pig model, which is an ideal alternative model for humans (Heinritz et al., 2013).

Previous studies shown that *C.B* addition alone or corn barn addition alone both have no significant effects on ADG and ADFI (Liu et al., 2018; Zhang et al., 2018). Here, the combination of *C.B* and corn bran keep the consistent effects on them, indicating the combination has no negative effects on pig growth. However, we noticed that separate addition of these two substances both reduced the specific microbial flora in pigs, especially for *C.B* addition on day 28 (Liu et al., 2018; Zhang et al., 2018). However, the combination application of them showed different effects. Herein, increase of microbial diversity motivated our interest to study their combination how to affect intestinal microbiota structure in present study.

Microbial changes caused by *C.B* and corn bran should be discussed separately by period. On day 14 after weaning, both within- and between-habitat diversity of fecal samples remained stable. As for specific alterations in the microbial community, reduced Erysipelotrichaceae in the *C.B* and Bran group, suggests a positive effect of corn barn and a reduced potential for erysipelas infection (Ding et al., 2015). The intestinal microbial structure of newly weaned pigs is immature, and not firmly established. So, weaning stress can easily disturb the dynamic balance of intestinal microbiota (Chen L. et al., 2017, 2018). In present study, lack of difference in the intestinal microbiota between treatments on day 14 may due to successful establishment of *C.B* in the early period after weaning (Zhang et al., 2018). Also, intestinal function is not mature enough to successful digest dietary fiber (Xu et al., 2014). Thus, if we want to investigate the additive effects of corn bran on intestinal microbiota, select of the appropriate period is essential.

Since diversity is considered as an indicator of healthy microbiota (Salonen et al., 2012), the increased diversity from day 14 to 28 suggests at least 28 days are required for the gut to adapt to weaning stresses (i.e., change in diet, social structure, and environment). Significant changes of microbial composition on day 28 indicated modulation of corn bran mainly occurred in the later period after weaning when a relatively stable microbial community has been established (Zhao et al., 2018). Addition of corn bran with high concentration of IDF provided fermentation substrates for intestinal microbiota such as *C.B* and increased the amount of unique OTUs and microbial variance. The present increase of Firmicutes has been proven to ferment polysaccharides to SCFAs (D’hoe et al., 2018) and orders of Clostridiales and Lactobacillales also showed a great boost in cellulose degradation. Clostridiales in the intestinal mucosa is a pivotal mediator for fiber fermentation, butyrate production, and mucosal immunity (David et al., 2015; Bensoussan et al., 2016). In addition, some bacteria of Clostridium such as *Ruminococcus flavefaciens*, *Ruminococcus bromii*, and *Faecalibacterium prausnitzii* can use the cellulosome system to degrade cellulose (Zhang et al., 2015; Hu et al., 2016). Among the Clostridiales, Ruminococcaceae increases in prevalence in diets enriched in resistant starch, while Lachnospiraceae is improved in a diet rich in wheat bran (Louis et al., 2014). In the *C.B* + Bran group, the higher proportion of Ruminococcaceae and Lachnospiraceae suggested an elevated demand for fiber degradation. Additionally, Ruminococcaceae and Lachnospiraceae are associated with lean phenotypes (Menni et al., 2017), which is consistent without performance effects in *C.B* + Bran group. When considering bacterial function, several changes merit attention. Prevotellaceae is reported to be associated with several human diseases, such as asthmatic airway inflammation and arthritis (Scher et al., 2013; Clarke et al., 2014). So, for humans, Prevotellaceae is thought to be an opportunistic pathogen (Lukens et al., 2014; Pianta et al., 2017). Decreased Prevotellaceae in the *C.B* + Bran group showed the benefits of combining *C.B* with corn bran. In sum, the addition of corn bran optimized the intestinal microbiota with increasing fiber-degrading bacteria and decreasing pathogens.

The wave of microbial fermentation in the intestine caused by the combination of *C.B* and corn bran deserves attention. Intestinal production of SCFAs depends on composition of intestinal microbes, substrate source and chyme transit time (Wichmann et al., 2013). In this study, fecal samples were used for SCFAs analysis. Unlike chyme samples, feces mainly reflect the nutritional difference between production and consumption. In the present study, we found that fecal acetate content declined with the *C.B* + Bran treatment on day 14 but increased on day 28. Acetate is the most abundant SCFA, and its concentration in the lumen is influenced by dynamic balance of production, use, and mucosal uptake (Elamin et al., 2013; Louis et al., 2014). Food with low viscosity such as bran could alter the intestinal microenvironment with reduced activity of amylase in small intestine (Desai et al., 2016; Martens, 2016), which could explain the decreased concentration of SCFAs on day 14. However, on day 28 the increased anaerobic bacteria in the *C.B* + Bran treatment, such as Ruminococcaceae and Lachnospiraceae are known to produce acetate and suppress the growth of Bacteroidales which is the preferential producer for propionate (Flint et al., 2008). Butyrate serves as a major energy source for intestinal enterocytes and exerts health-promoting effects on the colon (Huang et al., 2015). Bacteria synthetizes butyrate through two primary pathways. One pathway is a conversion of acetate to butyrate via butyryl-CoA (Duncan et al., 2002; Besten et al., 2013; Louis et al., 2014). The second pathway is a direct synthesis via butyrate kinase. *Lactobacillus*, *Megasphaera*, *Blautia*, and *Prevotella* are considered to participate in the butyrate producing (Berni Canani et al., 2016; Zhang et al., 2018). Among that, *Lactobacillus* was thought contact with butyrate production via expands butyrate-producing bacterial strains, like *Blautia*, *Roseburia*, and *Coprococcus* (Berni Canani et al., 2016). But here, the fluctuation of proportion in *Megasphaera*, and *Prevotella* made it is difficult to contact them with butyrate production. In present study, we have not observed any significant change in butyric acid content on day 14 and day 28, despite content of fecal butyrate increased slightly on day 28 along with increasing of fecal acetate. These results were consistent with previous study (Liu et al., 2018; Zhang et al., 2018). It should explains two things. First, the relationship of SCFAs concentrations in digesta and in feces should not be positively associated (Fan et al., 2017). Moreover, these results reminded us that producing butyrate maybe not the main ways of *C.B* or corn barn on improving intestinal environment. The specifically mechanism need further been illuminated.

Given our results, the effects of combination of *C.B* and corn bran should lie in providing substrates for intestinal fermentation, increasing acetate to reduce colonic pH, and optimizing intestinal microbiota which suppressed harmful bacteria.

## Conclusion

Addition of corn bran to *C.B* changed the intestinal microbial community greatly with increasing fiber-degrading bacteria including Ruminococcaceae and Lachnospiraceae and decreasing pathogens such as Erysipelotrichaceae and Prevotellaceae. IDF in the corn bran provided fermentable substrates for colonic microbiota and enhanced intestinal fermentation with elevated acetate content in feces on day 28. Thus, the combination of *C.B* and corn bran enhanced the benefits of the single addition of *C.B* with optimized intestinal microbiota and fermentation in the later period after weaning. Additionally, it suggested a new application for the use of corn bran as with synbiotics.

## Author Contributions

XM conceived and designed the research. JZ, JS, and JbZ conducted the research. JZ wrote the manuscript and analyzed the data. JS and XC wrote a part of manuscript and assisted in analysis of data. CN, WG, and LL contributed to sample analysis. TH, YC, JsZ, and XM critically reviewed the manuscript. LJ contributed to language review. All authors read and approved the final manuscript.

## Conflict of Interest Statement

The authors declare that the research was conducted in the absence of any commercial or financial relationships that could be construed as a potential conflict of interest.

## Footnotes

1 http://drive5.com/uparse/

2 http://rdp.cme.msu.edu/

3 http://qiime.org/scripts/alpha_rarefaction.html
